# Pacific Broad Tapeworm *Adenocephalus pacificus* as a Causative Agent of Globally Reemerging Diphyllobothriosis

**DOI:** 10.3201/eid2110.150516

**Published:** 2015-10

**Authors:** Roman Kuchta, Marcus Enrique Serrano-Martínez, Tomas Scholz

**Affiliations:** Institute of Parasitology, Biology Centre of the Czech Academy of Sciences, České Budějovice, Czech Republic (R. Kuchta, T. Scholz);; Faculty of Veterinary Medicine, Cayetano Heredia University, Lima, Peru (M.E. Serrano-Martínez)

**Keywords:** Pacific broad tapeworm, Cestoda, *Adenocephalus pacificus*, *Diphyllobothrium pacificum*, cestodiasis, cestodosis, diphyllobothriid cestodes, fish-borne disease, enteric infections, helminth, parasites, endoparasite, parasitoses, zoonoses, Pacific Ocean, South America

## Abstract

The Pacific broad tapeworm *Adenocephalus pacificus* (syn. *Diphyllobothrium pacificum*) is the causative agent of the third most common fish-borne cestodosis among humans. Although most of the nearly 1,000 cases among humans have been reported in South America (Peru, Chile, and Ecuador), cases recently imported to Europe demonstrate the potential for spread of this tapeworm throughout the world as a result of global trade of fresh or chilled marine fish and travel or migration of humans. We provide a comprehensive survey of human cases of infection with this zoonotic parasite, summarize the history of this re-emerging disease, and identify marine fish species that may serve as a source of human infection when eaten raw or undercooked.

## Abstract 2

Certain types of marine fish, eaten raw or undercooked, may serve as a source of human infection with these parasites.

Infection with the tapeworm *Adenocephalus pacificus* (syn. *Diphyllobothrium pacificum*) (Cestoda: Diphyllobothriidea) was described by Nybelin in 1931 in the Juan Fernández fur seal, *Arctocephalus philippii*, from waters of the Juan Fernández Islands off the coast of Chile. This parasite has been reported among 9 of 16 species of extant otariid seals and has wide distribution, mostly in the Southern Hemisphere (*1*). The convoluted taxonomic history of the genus, which was synonymized with *Diphyllobothrium* Cobbold, 1858, has been recently reviewed by Hernández-Orts et al. (*1*), who resurrected the name *Adenocephalus* Nybelin 1931, on the basis of molecular and morphological evidence and transferred *D. pacificum* back to *A. pacificus*. However, in this article, we use the established term “diphyllobothriosis” to describe infection with parasites in this genus and for human disease caused by *A. pacificus*. 

In addition to otariids, infections with *A. pacificus* have been reported among humans and dogs who consumed raw or undercooked marine fishes (*2*). The first 2 human cases of diphyllobothriosis caused by this species were briefly reported from Callao, Lima, Peru, in 1957 (*3*). Another case in a student from Trujillo, Peru was erroneously reported as having been caused by *Diphyllobothrium latum* (Linnaeus, 1758) (*4*). However, the eggs of this tapeworm were found in coprolites and mummified humans in several archeological sites in Peru and northern Chile (*5*).

So far, ≈50 records of diphyllobothriosis caused by *A. pacificus* have been published; many of them were published in regional journals that are difficult to obtain (Technical Appendix Table 1). Neither a synthesis of these cases nor an exhaustive list of fish that are potential intermediate hosts has been published. We conducted an extensive search of the literature and examined extensive samples of *A. pacificus* (*1*,*6*) to present a comprehensive synopsis of the human disease caused by *A. pacificus*, including data on the geographic distribution of human cases and a survey of potential fish hosts of this zoonotic parasite that serve as a source of human infection.

## History of *Adenocephalus pacificus* Diphyllobothriosis among Humans

### Archeological Data

The Pacific broad tapeworm *A. pacificus* seems to have co-existed with humans at least since the early Neolithic period, as evidenced by the recovery of diphyllobothriidean eggs in archeological samples such as coprolites or mummies (*5*). The first findings of cestode eggs in coprolites from South America were identified as those of *Diphyllobothrium* sp., *D. latum*, or *D. trinitatis* Cameron, 1936 (*5*). However, this species identification is questionable, because evidence shows that *D. latum* originally occurred in the Northern Hemisphere only (*2*); *D. trinitatis* is a *species inquirenda* (i.e., a species of uncertain taxonomic status because of insufficient available data).

**Figure 1:**
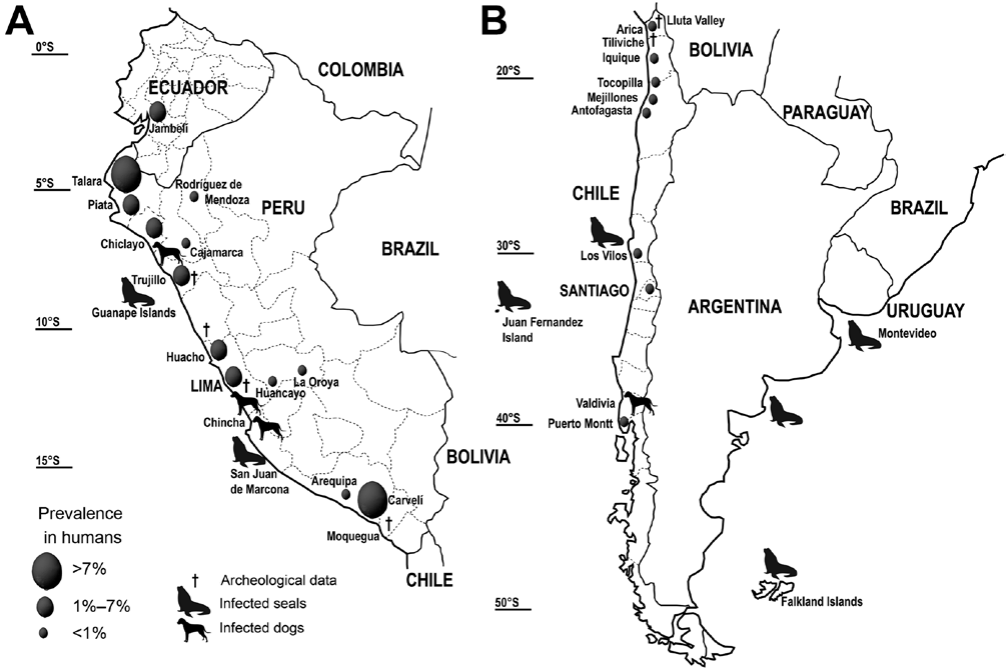
Distribution of the Pacific broad tapeworm *Adenocephalus pacificus* among humans and wild animals on the A) northern and B) southern Pacific coast of South America.

The first archeological records of *A. pacificus* found in coprolites were from the coastal site of Los Gavilanes in Peru, dated from 2850 to 2700 bce (*7*), and from the site of Tiliviche in northern Chile (Iquique), with Chinchorro culture dating from 4110 to 1950 BCE (*8*) (Figure 1). The latter site lies 40 km from the Pacific coast at an altitude of 950 meters, which may demonstrate that diphyllobothriosis was not limited to the coastal areas, either as a result of import of marine fish or movement of infected persons from the coast (*8*,*9*). The presence of *A. pacificus* eggs were then confirmed in other mummified bodies of humans in Chinchorro, dated 3050–2050 bce (*10*). (Figure 1).

The oldest record of *A. pacificus* from the South American continent was dated to the Neolithic period, as long ago as 8000 bce, from an unknown locality on the northern Peruvian coast (*11*), but this dating later appeared to be incorrect because radiocarbon dates were not precise (K. Reinhard, pers. comm.). The eggs of the Pacific broad tapeworm were reported from Preceramic cultures in Peru (2850–2500 bce) in Huarmey Valley and Huaca Prieta (*7*), as well as from Ceramic cultures (*5*). Several records are also known from the pre-Inca (Chiribaya culture, 800–1400 bce) and Inca (1476–1534 bce) eras in Peru and northern Chile (*5*) (Figure 1). A comparison of data from the pre-Inca and Inca eras in San Geronimo, Chile, indicated that the number of cases among humans increased when Incas conquested this region (*12*).

The archeological record of eggs of *A. pacificus* in the skeleton of a child found on Adak Island in Alaska, reported by Bouchet et al. (*13*), is unlikely accurate because the shape of the egg does not correspond to that typical of *A. pacificus*. Some *Diphyllobothrium* species may have been misidentified (*2*).

### Modern Times and the Present

No reliable reports of diphyllobothriosis are available for most of the modern or post-colonial period, after 1500 ce (*5*). The first confirmed case of human infection with *A. pacificus* in modern times was identified in 1967 by Baer (*14*), who disputed a previously published report of a tapeworm misidentified as *D. latum* by Miranda et al. (*4*) and reported an additional 7 cases (Technical Appendix Table 1). Also in 1967, Rêgo (*15*) published a case report of a student from Peru who was in Argentina and was infected with a tapeworm identified as *Lueheella* sp. (syn. of *Spirometra*). Re-examination of voucher specimens from the Helminthological Collection of the Instituto Oswaldo Cruz in Rio de Janeiro, Brazil (CHIOC nos. 30161 and 30162) revealed that the tapeworm was misidentified and was actually *A. pacificus* (R. Kuchta and T. Scholz, unpub. data).

Since the 1950s, ≈1,000 cases of human infection with *A. pacificus* tapeworms have been reported. These reports are chronologically summarized in the Technical Appendix Table 1, together with all published records of *A. pacificus* adults from all species of definitive hosts.

The number of reported cases during 1957–2015 in individual decades fluctuated irregularly, partly depending on whether comprehensive research reports of numerous human cases or only individual case reports were published in a given decade (Technical Appendix Table 1). The number of human infections increased most considerably during 1981–1990. In the 21st century, the number of reported cases declined conspicuously, but this may be related to an inexplicable gap in reporting diphyllobothriosis in Latin America after 1990 rather than to an actual decline of human infections. Diphyllobothriosis is not considered to represent a serious health problem in Peru, especially when its effect on human health is compared to that caused by cysticercosis, which is widely distributed in that country. 

## Distribution of Cases among Humans

*A. pacificus* tapeworms are the most widely distributed endoparasitic helminth of seals, and infections occur in temperate areas of the North and South Pacific regions and in some southern temperate zones of the Atlantic and Indian oceans (*1*) (Technical Appendix Table 1). In contrast, human infections have been reported almost exclusively from the Pacific coast of South America, mainly from Peru and, in a relatively few records, from Chile and Ecuador (Figure 1).

### South America

*A. pacificus* is the most common cestode species that causes fish-borne diseases in South America (*2*). Other diphyllobothriid species, such as *D. latum*, and sporadically, *D. dendriticum* (Nitzsch, 1824), have been rarely reported as adults in human infections or as plerocercoids (larvae) from fishes in Chile, Argentina, and Brazil. Identification was verified by molecular data for only 3 cases of *D. latum* infection (*16*).

### Peru

On the coast of Peru, ≈1,000 cases of infection with *A. pacificus* tapeworms have been reported since 1957 (*17*) (Technical Appendix Table 1). Prevalence has been as high as 7.5% in some regions but is ≈2% in most regions (*18*,*19*) (Figure 1). Some studies showed prevalence of up to 83% (*20*), but these data were calculated on the basis of small sample sizes. 

Most cases are associated with the coast, but some have been reported from inland provinces such as Amazonas and Junín (Figure 1; Technical Appendix Table 1). One case was reported from a town in the Andean region at an altitude of 3,460 m (*21*).

### Other Countries in South America

Only 18 cases of human infection with *A. pacificus* tapeworms have been reported from Chile since 1975 (*16*,*22*) (Technical Appendix Table 1), most from Antofagasta in northern Chile. One case of uncertain origin was reported from Los Lagos in Puerto Montt, located in southern Chile (*23*) (Figure 1). The cases from Chile were proposed to be related to the *El Niño* Southern Oscillations (ENSO) phenomenon, presumably caused by changes in water temperatures that result in the southern displacement of marine fish native to Peruvian waters and the creation of conditions favorable for the overgrowth of copepods (*22*,*24*). However, no evidence supports this theory, and reports of human cases do not correspond to the years of the ENSO phenomenon (Technical Appendix Table 1).

A few cases were also reported from Ecuador, where the northernmost case among humans (latitude 3°S) was diagnosed (Figure 1). However, only 1 epidemiologic study reporting diphyllobothriosis is available (*25*); of 373 fecal samples examined, 13 (3.5%) were infected.

### Outside South America

Few records exist of *A. pacificus* infections in humans outside South America. Cases among 6 humans were reported from Japan (*26*; Technical Appendix Table 1), but these cases have not been confirmed by molecular data. In Japan, as many as 11 species of diphyllobothriid cestodes have been reported to infect humans (*2*), and misidentification with other species cannot be ruled out. The first case was described by Kamo et al., who examined tapeworms found by Sunagawa in a man, 35 years of age, from Okinawa Prefecture (*27*). Another case may have been imported: the infected person, a seaman from Kitakyushu City, served as a crew member on trips along the coast of Africa (Technical Appendix Table 1). The most recent human case reported in Japan was diagnosed in a man from Matsuyama City, Ehime Prefecture (*26*). *A. pacificus* was also reported in fur seals in Japan (*28*), but all reported human cases are limited to southern Japan (Okinawa, Kyushu, Shikoku), which is outside of the area of distribution of fur seals. The origin of human infections in Japan is thus unclear.

The distribution area of *A. pacificus* tapeworms among otariids is much wider than that in humans, which apparently represent incidental, atypical definitive hosts. The tapeworm is distributed globally, documented by confirmed records from the North Pacific (Canada, Far East Asia, United States), South Pacific (Peru, Chile, New Zealand), South Atlantic (Argentina, South Africa, Uruguay), and South Indian (Australia) Oceans (*1*) (Technical Appendix Table 1). However, very few autochthonous human cases have been reported from the northern hemisphere. 

In 1937, Rutkevich described 2 new species of *Diphyllobothrium*: *D*. *giljacicum* and *D. luxi*, from the Nivkh people on the Sakhalin Island (Far East Russia), collected during expedition in 1928 (*29*). *D. luxi* is most probably the synonym for *D*. *nihonkaiense*; however, *D*. *giljacicum* described from 10 Nivkh (also known as Gilyak) people on the west coast of the Sakhalin Island seems to be closely related to *A.* *pacificus*. The longest specimen was 3.63 meters, and the worm showed several similarities with *A*. *pacificus*: shape of bothria and scolex, wide and short neck, separated opening of cirrus-sac and vagina, and small eggs (<57 μm; eggs of *D*. *nihonkaiense* and *D. latum* are usually >60 μm). Both species described by Rutkevich (*29*) were incorrectly synonymized as *D*. *latum* (*30*). With exception of the sporadic cases from the Sakhalin Island (*29*), there are no records of *A. pacificus* infection in humans in North America and Far East Asia**,** even though fur seals are heavily infected with this cestode on the northern Pacific coasts of these continents (*1*) and other diphyllobothriid cestodes such as *D. nihonkaiense* occur in man relatively frequently (*2*).

## Recent Cases among Humans

The ability of the *A. pacificus* tapeworm to expand its distribution area globally is demonstrated by infections of humans in Spain, which have recently been confirmed by molecular data (*6*,*31*). The source of human infection in Europe remains to be clarified, but commercial import of marine fish stored on ice from areas to which the parasite is endemic, such as Chile or Ecuador, may be a plausible explanation. Spain is the third largest importer of fish and seafood in the world and imports fresh or chilled fish (i.e., those that may harbor infective plerocercoids of diphyllobothriid tapeworms) (*2*). The import of fish products from South America is critical in the spread of the parasite; countries to which *A. pacificus* tapeworm is endemic (i.e., Ecuador, Chile, and Peru) represent major exporters (*6*). Travel-associated cases or migration of humans may also result in distribution of diphyllobothriid cestodes to area outside endemic zones.

## Source of Human Infection

**Figure 2:**
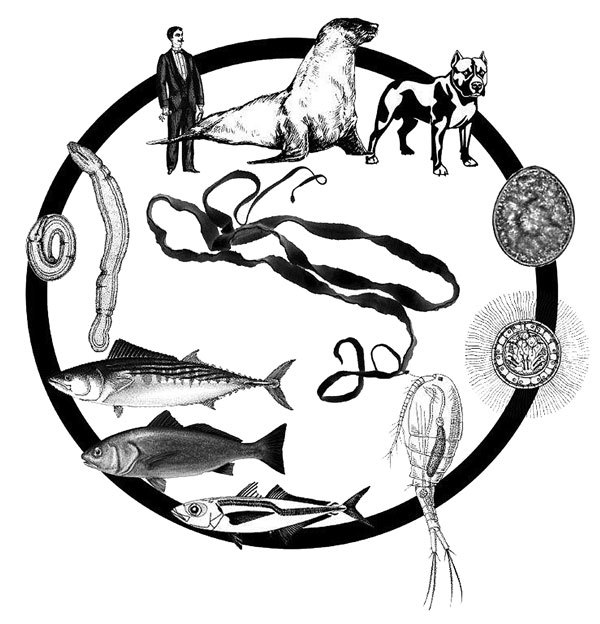
Life cycle of the Pacific broad tapeworm. From top: definitive hosts (otariid seals, humans, dogs); egg; coracidium; potentional first intermediate host (copepod); second intermediate hosts (*Sarda chiliensis*, *Sciaena deliciosa*, *Trachurus murphyi*); encysted plerocercoids in body cavity of fish* Adenocephalus pacificus*.

The life cycle of *A. pacificus* tapeworms is not completely known, and no data on the first intermediate hosts are available. Because marine mammals serve as definitive hosts, the life cycle is undoubtedly completed in the sea, unlike the freshwater cycle of most other human-infecting diphyllobothriids (*2*). Thus, we may assume that the cycle includes marine copepods as the first intermediate hosts, marine fish as the second intermediate hosts, and fish-eating mammals, including humans, as the definitive hosts (*2*) (Figure 2).

Humans become infected with *A. pacificus* tapeworms when they eat raw or insufficiently cooked marine fish or food items made from these fish. In coastal regions of Peru, dishes made with raw fish, such as cebiche, tiradito, and chinguirito, are popular and represent the main source of human infections (*2**,**32*). Several marine fish inhabiting waters off the Peruvian coast have been reported as potential intermediate hosts of *A. pacificus*, but their actual spectrum has never been critically reviewed.

**Figure 3:**
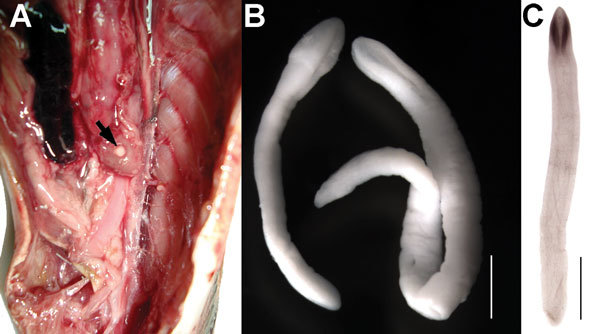
Photomicrograph of plerocercoids of *Adenocephalus pacificus* from *Sarda cholensis* off Peru. A) Body cavity with encysted plerocercoid (arrow). B) Liberated plerocercoids under stereomicroscope. Scale bar indicates 1 mm. C) Whole mount of the plerocercoid. Scale bar indicates 2 mm.

The plerocercoids of *A*. *pacificus* are encysted in membraneous cysts in the viscera, on the peritoneum or in the stomach wall; some have also been found outside of the intestinal wall and in the gonads (*32*) (Figure 3). However, they have never been found in musculature. The cysts are thin-walled, oval, pearly white, and measure 2–4 mm in diameter (*33*,*34*). Excysted plerocercoids are relatively large (total length of 4–22 mm), and their anterior end (future scolex) possesses distinct bothria measuring 0.5–1.4 mm in length (Figure 3); the surface of plerocercoids is wrinkled and covered with microtriches ≈4 µm long. The species identification of these plerocercoids as *A*. *pacificus* was confirmed by sequencing of the *cox1* gene (*1*). 

Baer (*33*) first reported plerocercoids of *A. pacificus* from 2 species of marine fish caught on the coast of Peru: the Eastern Pacific bonito *Sarda chiliensis* and Atlantic Spanish mackerel *Scomberomorus maculatus*. However, the first morphological description of plerocercoids supposedly belonging to *A. pacificus* was made by Tantalean (*33*), who found plerocercoids in the peritoneum and gonads of the lorna drum, *Sciaena deliciosa,* and the Peruvian banded croaker, *Paralonchurus peruanus*. Plerocercoids of other diphyllobothriid cestodes may also use marine fish as intermediate hosts (*33*). To date, plerocercoids allegedly from *A. pacificus* tapeworms were found in 21 fish species of 12 phylogenetically unrelated and ecologically distant families of different orders, including 1 shark species (Technical Appendix Table 2). However, only 8 fish species were confirmed as suitable second intermediate hosts of *A. pacificus* by experimental infections of dogs or genotyping (Technical Appendix Table 2). Other fish species may serve as second intermediate hosts, as can be assumed from anamnestic data of humans infected with *A. pacificus* tapeworms (Technical Appendix Tables 2, 3), but their actual role in transmission must be confirmed by finding *A. pacificus* plerocercoids. Documented prevalence of fish infection with *A. pacificus* plerocercoids has seldom exceeded 20% (Technical Appendix Table 2). We dissected 79 fish of 5 species collected off the coast of Lima, Peru and found 66 plerocercoids in the body cavity of 2 species with intensity of 2–3 per fish (Technical Appendix Table 2, Figure 3). 

## Pathology and Clinical Signs

Diphyllobothriosis is notoriously known as a potential cause of vitamin B12 avitaminosis and megaloblastic anemia (*35*). However, this effect of the parasite on its human host is rare, and most cases in which these conditions were reported as human infections with *D. latum* tapeworms occurred in Finland after World War II (*2*). Clinical symptoms of diphyllobothriosis are usually mild; the most common clinical signs are abdominal discomfort or pain and diarrhea (*2*).

Clinical signs related to human *A. pacificus* infection are poorly known and have been studied in more detail only 3 times, all in Peru: Lumbreras et al. (*36*) studied 32 cases, Medina Flores et al. (*17*) 21 cases, and Jiménez et al. (*37*) 20 patients. Additionally, 37 individual symptom reports have also been analyzed (Technical Appendix Table 3). From a total of 110 case-patients, 18 had no clinical signs, but most of the symptoms were mild or nonspecific, such as abdominal pain (n = 74), diarrhea (n = 37), weight loss (n = 17), nausea (n = 11), or vomiting (n = 5) (Technical Appendix Table 3). Megaloblastic anemia and vitamin B12 deficit were reported in 1 and 5 patients, respectively (*36*–*38*).

Typically, *A. pacificus* infections are registered after spontaneous elimination of tapeworms from the patient (Technical Appendix Table 3). Diphyllobothriosis caused by *A. pacificus* infection has sporadically reported in AIDS patients; García et al. (*39*) found only 4 (2%) of 217 AIDS patients infected with this tapeworm, but diarrhea may be a consequential complication and causes malabsorption and malnutrition among these patients.

## Diagnosis and Control

Differential diagnosis of diphyllobothriid cestodes from human-infecting species of *Taenia* is easy and straightforward because they differ by the position of gonopores (median in diphyllobothriids versus lateral in taeniids). In contrast, identification of most diphyllobothriid cestodes from clinical material is usually impossible based only on their morphologic characteristics (*2*). The *A. pacificus* tapeworm represents one of the few exceptions because its proglottids possess papilla-like protuberances separated by semicircular pits between the genital atrium and the anterior margins of segments (*1*); these protuberances are absent in other species that cause diphyllobothriosis. In addition, *A. pacificus* eggs are somewhat smaller and more spherical than those of human-infecting species of *Diphyllobothrium*, and the worm’s genital atrium has an almost equatorial position, which distinguishes it from *Diphyllobothrium*, in which it has a more anterior position (*1*,*6*). 

The only way to exactly determine the species of the causative agent and thus the origin of the infection is through sequencing and analysis of the parasites’ genes. To facilitate differential identification of morphologically indistinguishable human-infecting broad fish tapeworms (*D. latum*, *D. dendriticum*, *D. nihonkaiense*) and *A. pacificus* in clinical samples, a diagnostic method has been developed and optimized by Wicht et al. (*40*). The method is based on results of a multiplex PCR amplification of a selected gene (*cox1*) and does not involve sequencing; thus, this method represents a substantively less costly and easily interpretable approach to be used routinely, mainly by medical diagnostic laboratories.

Treatment of patients who have diphyllobothriosis is simple and highly effective by a single dose of niclosamide (2 g in adults) or praziquantel (*2*,*36*). Lumbreras et al. (*3*^6^) sufficiently treated 32 case-patients by using a single dose of 10 mg/kg of praziquantel. However, a single administration of a 25–50 mg/kg dose is usually applied to ensure complete expulsion of diphyllobothriid tapeworms (*2*).

The imports of fishery products are subject to official certification. The national authorities must also guarantee that the relevant hygiene and public health requirements are met. The provisions are aimed at ensuring high standards and at preventing any contamination of the product during processing. Scholz et al. and Kuchta et al. compiled information for processing fish to avoid survival of plerocercoids of diphyllobothriid cestodes (*2*).

## Conclusions

Human disease caused by infection with the Pacific broad tapeworm *A*. *pacificus* is endemic to the Pacific coast of South America, and most (>99%) clinical cases are reported from Peru. However, this tapeworm species occurs globally, and recent cases of human infection in Europe illustrate that more attention should be paid to this emergent fishborne zoonosis (*6*). The increasing popularity of eating raw or undercooked fish, import of fresh chilled or insufficiently frozen fish, and traveling and migration of humans represent risk factors that may contribute to a more global expansion of fishborne parasitoses caused by diphyllobothriid cestodes, including *A. pacificus*.

Samples of tapeworms found in humans should be processed adequately to enable molecular diagnosis and thus identification of the sources of human infection and the geographic origin of parasite infective stages (plerocercoids). Therefore, positive fecal samples or pieces of the strobila should be placed immediately to 96%–99% molecular-grade ethanol (i.e., not technical, denaturated ethanol). Samples should never be fixed with formalin unless part of the same sample is also fixed with ethanol. Fixed samples should be sent to a specialized parasitological laboratory, in which molecular and morphological identification can be performed. The laboratory of the Institute of Parasitology, Biology Centre of the Czech Academy of Sciences, České Budějovice, Czech Republic, is able to analyze and reliably identify clinical samples of diphyllobothriid cestodes free of charge. We highly recommend that representative samples be deposited in a parasite collection so that specialists can conduct further study if necessary.

For a better control of zoonotic disease caused by the Pacific broad tapeworm, gaps in our knowledge of its biology, epidemiology, and transmission should be filled. In particular, a limited knowledge of the fish intermediate hosts impedes a more effective control of fishery products and thus restriction of export of those fish that may harbor *A. pacificus* plerocercoids*.* Additionally, little is known about the factors that have contributed to the almost complete absence of human diphyllobothriosis outside South America, especially in the North Pacific, where *A. pacificus* tapeworms occurs frequently in fur seals but no human cases have been confirmed. The use of molecular markers for reliable identification of clinical samples should become an obligatory practice because it is necessary for a better understanding of the epidemiology of this zoonotic parasite.

**Technical Appendix.** Historical survey of records of adult *Adenocephalus* *pacificus*, marine fish reported as second intermediate hosts of *A. pacificus*, and descriptions of clinical cases of diphyllobothriosis caused by *A. pacificus*.
