# Supplementary material for: Pacific Broad Tapeworm Adenocephalus pacificus as a Causative Agent of Globally Reemerging Diphyllobothriosis
Source: Emerg Infect Dis. 2015 Oct;21(10):1697–703. doi: 10.3201/eid2110.150516 (PMC4593442; doi:10.3201/eid2110.150516)
Supplement: Supplementary file 1 — Technical Appendix. Historical survey of records of adult Adenocephalus pacificus, marine fish reported as second intermediate hosts of A. pacificus, and descriptions of clinical cases of diphyllobothriosis caused by A. pacificus. [file 15-0516-Techapp-s1.pdf]

Article DOI: http://dx.doi.org/10.3201/eid2110.150516

Pacific Broad Tapeworm *Adenocephalus pacificus* as a Causative Agent of Globally Re-emerging Diphyllobothriosis

Technical Appendix

Technical Appendix Table 1. Historical survey of global records of adult *Adenocephalus pacificus* tapeworm*

Year	Name	Host	Location	Reference
1899	*Bothriocephalus* sp.	*Callorhinus ursinus*	St. George Island, Alaska, USA	(*1*)
1931	*Adenocephalus pacificus* n. sp.†	*Arctocephalus australis*	Juan Fernandez Island, Chile	(*2*)
	Adenocephalus septentrionalis n. sp.‡	*Callorhinus ursinus*	St. George Island, Alaska	
1937	*Diphyllobothrium arctocephali* n. sp.	*Arctocephalus pusillus*	Lady Julia Percy Island, Australia	(*3*)
	D. arctocephalinum	*Arctocephalus forsteri*	Pearson Island, Australia	(*4*)
1941	*A. septentrionalis*	*Callorhinus ursinus*	Commander Islands, Russia	(*5*)
1947	*Cordicephalus arctocephalinus*	*Callorhinus ursinus*	St. Pauls Island, Alaska, USA	(*6*)
1948	Species No. 2	*Callorhinus ursinus*	St. Pauls Island, Alaska, USA	(*7*)
1951	*A. pacificus*	*Callorhinus ursinus*	Onahama, Japan	(*8*)
1952	*D. hians*	*Callorhinus ursinus*	Sakhalin, Russia	(*9*)
	D. glaciale	*Callorhinus ursinus*	Pribilof Island, Alaska, USA	(*10*)
	D. scoticum	*Otaria flavescens*	Falkland Island, UK	(*10*)
1954	*D. glaciale*	*Callorhinus ursinus*	Pribilof Island, Alaska, USA	(*11*)
1955	*Diphyllobothrium krotovi* n. sp.	*Callorhinus ursinus*	Tyuleniy Island, Russia	(*12*)
1956	*D. pacificum*	*Eumetopias jubatus*	Triangle Island, Canada	(*13*)
**1957**	***D. latum***	**Human (2 cases)**	**Lima, Peru**	(*94*)
**1961**	***D. latum***	**Human (1 case)**	**Trujillo, Peru**	(*14*)
**1962**	***D. latum***	**Human (1 case)**	**Chiclayo, Peru**	(*15*)
**1963**	***D. latum***	**Human (1 case)**	**La Oroya, Peru**	(*67*)
**1964**	***D. latum***	**Human (1 case)**	**Lima, Peru**	(*67*)
	D. latum	**Human (1 case)**	**Lima, Peru**	(*67*)
**1965**	***D. latum***	**Human (5 cases)**	**Lima, Peru**	(*67*)
	D. latum	**Human (1 case)**	**Trujillo, Peru**	(*67*)
	D. latum	**Human (1 case)**	**Chiclayo, Peru**	(*67*)
	D. latum	**Human (2 cases)**	**Lima, Peru**	(*67*)
	D. pacificum	*Callorhinus ursinus*	Pribilof Island, Alaska, USA	(*16*)
**1967**	***D. pacificum***	**Human (7 cases)**	**Trujillo, Peru**	(*17*)
	D. latum	**Human (15 cases)**	**Lima, Peru**	(*67*)
	D. latum	**Human (3 cases)**	**Trujillo, Peru**	(*18*)
	***Luehella* sp.**	**Human (1 case)**	**Peruvian student in Argentina**	(*19*)
1968	*Diphyllobothrium atlanticum* n. sp.	*Arctocephalus pusillus*	off South Africa	(*20*)
1969	*D. pacificum*	*Otaria byronica*	Guañape Islands, Peru	(*21*)
	D. pacificum	*Callorhinus ursinus*	Off northern Japan coast	(*22*)
1970	*D. glaciale*	*Eumetopias jubatus*	Off California coast, USA	(*23*)
1971	*D. krotovi*	*Callorhinus ursinus*	Tyuleniy Island, Russia	(*24*)
1975	*D. krotovi*	*Callorhinus ursinus*	Kuril Islands	(*25*)
	D. pacificum	**Human (2 cases)**	**Salaverry, Peru**	(*67*)
	D. pacificum	**Human (11 cases)**	**Arequipa, Peru**	(*67*)
	D. pacificum	**Human (total 115 cases)**	**Peru**	(*67*)
**1976**	***D. pacificum***	**Human (1 case)**	**Los Vilos, Chile**	(*26*)
	D. latum	**Human (1 case)**	**Valdivia, Chile**	(*27*)
	D. pacificum	**Human (2 cases)**	**Mejillones & Antofagasta, Chile**	(*28*)
1977	*D. pacificum*	*Otaria flavescens*	Los Vilos, Chile	(*29*)
	D. pacificum	**Human (25 cases)**	**Lima, Peru**	(*30*)
	D. pacificum	**Human (7 cases)**	**Lambayeque, Peru**	(*31*)
1978	*D. pacificum*	*Eumetopias jubatus*	off Oregon, USA	(*32*)
**1979**	***D. pacificum***	**Human (11 cases)**	**North Chile**	(*33*)
1980	*D. pacificum*	*Arctocephalus philippii*	Alejandro Selkirk Island, Chile	(*34*)
1981	*D. pacificum*	*Otaria flavescens*	Isla Santa Maria, Chile	(*35*)
	D. pacificum	*Callorhinus ursinus*	Northern Honshu, Japan	(*36*)
**1982**	***D. pacificum***	**Human (1 case)§**	**Okinawa, Japan**	(*37*)
	D. pacificum	**Human (32 cases)**	**Lima, Peru**	(*38*)
**1983**	***D. pacificum***	**Human¶**	**Los Gavilanes, Peru**	(*39*)
	D. pacificum#	Dog (*Canis familiaris*)	Valdivia, Chile	(*40*)
**1984**	***D. pacificum***	**Human¶**	**Tiliviche, Chile**	(*41*)
1986	*D. pacificum*	*Eumetopias jubatus*	Gulf of Alaska, USA	(*42*)

Year	Name	Host	Location	Reference
	D. pacificum	*Callorhinus ursinus*	Medny Island in the Bering Sea	(*43*)
**1987¶**	***D. pacificum***	**Human (1 case)**	**Japan (seaman)**	(*44*)
**1988**	***D. pacificum***	**Human (1 case)**	**Santiago de Chile, Chile**	(*45*)
1989	*Diphyllobothrium* sp.	*Arctocephalus tropicalis*	Gough Island, South Africa	(*46*)
**1990**	***D. pacificum***	**Human (1 case)**	**Huancayo, Juníh, Peru**	(*47*)
	D. pacificum	**Human (1 case)**	**seaman in Kyushu, Japan**	(*48*)
	D. atlanticum	*Arctocephalus pusillus*	Namibia	(*49*)
**1991**	***D. pacificum***	**Human (13 cases)**	**Jambeli archipelago, Ecuador**	(*50*)
1993¶	*Diphyllobothrium* sp.	*Callorhinus ursinus*	N California, USA	(*51*)
	D. pacificum	*Otaria flavescens*	Juan de Marcona, Peru	(*52*)
	D. pacificum	**Human (1 case)**	**Matsuyama City, Japan**	(*53*)
	D. pacificum	**Human (18 cases)**	**Arequipa, Peru**	(*67*)
**1994**	***D. pacificum***	**Human (13 cases)**	**Lima, Peru**	(*54*)
	D. pacificum	**Human (5 cases)**	**San Juan de Miraflores, Peru**	(*55*)
**1995¶**	***D. pacificum***	**Human (14 cases)**	**Paiján, La Libertad, Peru**	(*56*)
**1996**	***D. pacificum***	**Human (2 cases)**	**Lima, Peru**	(*57*)
**1997**	***D. pacificum***	**Human (597 cases)**	**Lima, Peru**	(*67*)
1998¶	*D. pacificum*	*Arctocephalus pusillus*	Off Cape coast of South Africa	(*58*)
	D. pacificum	*Callorhinus ursinus*	Medny Island in the Bering Sea	(*59*)
**1999**	***D. pacificum***	**Human (1 case)**	**Chile**	(*60*)
**2000**	***D. pacificum***	**Human (21 cases)**	**Peru**	(*61*)
	D. pacificum	**Human (1 case)**	**Antofagasta, Chile**	(*62*)
2001	*D. pacificum*	Dog (*Canis familiaris*)	Chincha Alta, Peru	(*63*)
	D. pacificum	**Human (3 cases)**	**Antofagasta, Chile**	(*64*)
	D. pacificum	**Human (790 cases)****	**Peru (several localities)**	(*65*)
	D. pacificum	**Human (1 case)**	**Trujillo, Peru**	(*82*)
**2002**	***D. latum***	**Human (1 case)**	**Spain**	(*66*)
	D. pacificum	**Human (21 cases)**	**Lima, Peru**	(*67*)
**2003**	***Diphyllobothrium* sp.**	**Human ¶**	**Ilo, southern Peru**	(*68*)
	D. pacificum	**Human ¶**	**Osmore drainage, Peru**	(*69*)
	D. pacificum#	**Human ¶**	**northern Chile**	(*70*)
	D. pacificum#	**Human ¶**	**Lluta Valley, Chile**	(*71*)
**2006**	***D. pacificum***	**Human (2 cases)**	**Lima, Peru**	(*72*)
	D. pacificum	*A. australis*, *O. flavescens*	Montevideo, Uruguay	(*73*)
**2010¶**	***D. pacificum***	**Human (2 cases)**	**Antofagasta, Santiago, Chile**	(*74*)
	D. pacificum & *A. arctocephalinum*	*C. ursinus*, *O. flavescens*	Japan & Juan Fernandez Island	(*75*)
**2011**	***D. pacificum***	**Human (1 case)**	**Arequipa, Peru**	(*76*)
**2012**	***D. pacificum***	**Human (20 cases)**	**Tumbles, Piura, Lima, Peru**	(*77*)
	Diphyllobothrium sp.	Dog§	Osmore river in southern Peru	(*78*)
2013	*Diphyllobothrium* sp.	*A. australis*, *O. flavescens*	northern Patagonia, Argentina	(*79*)
	D. pacificum	**Human¶**	**Peru and Chile**	(*80*)
	D. pacificum	**Human¶**	**Peru**	(*81*)
**2014**	***D. pacificum***	**Human (3 cases)**	**Spain**	(*83*)
	D. pacificum	**Human (1 case)**	**Spain**	(*84*)
2015	*A. pacificus*	Several hosts	Several localities	(*85*)

*Bold text indicates cases among human patients.
†New genus Adenocephalus established.
‡Described from material collected by Stiles and Hassall (1899).
§Re-examination of material published by Sunagawa (1965).
¶Archeological finding (coprolites or mummy).
#ENSO reported in 1983, 1987, 1992, 1995, 1998, 2003, and 2010.
**Report of all cases from 1981–2001 by Ministry of Health, Peru.

Technical Appendix Table 2. Marine fish reported in the South Pacific Ocean as second intermediate hosts of *Adenocephalus pacificus*,*

Host species and family	Site	Location	Prevalence	Reference
Anisotremus scapularis HA	v,p	Peruvian coast	ND	(*86*); (*87*)†; (*89*)† 1417
Ariopsis seemanni **AR**	**p**	**Trujillo coast**	**4/32**	**(*87*)**
Cilus gilberti SC	ND	Peru coast	ND	(*18*)‡
Coryphaena hippurus CO	v,p	Peru coast	ND	(*77*‡, *88*)
Cynoscion analis SC	v	Trujillo coast	2/43	(*89*)
Galeichthys peruvianus **AR**	**v,p**	**Peru coast**	**16/30**	**(*90*), PS**
Genypterus maculates OP	v	Trujillo coast	1/6	(*89*)
Menticirrhus ophicephalus SC	ND	Peru coast	ND	(*86*)
Merluccius gayi peruanus ME	p	Trujillo coast	2/32	(47) ‡ (*86*,*89*),
Mugil cephalus MU	ND	Peru coast	ND	(*17*,*18*,*77*)‡ (*91*); † 50 (*89*); † 46 (*87*); † 23; PS† 10
Mustelus lunulatus TR	ND	Trujillo, Peru	ND	(*18*)‡
Paralichthys adspersus **PA**	**s**	**Trujillo coast**	**2/27**	**(*87*)**
Paralonchurus peruanus SC	i	Chimbote, Peru	1/180	(*91*); (*17, 18, 67, 77*)‡; (*21*) † 27; (*89*)† 24
Sarda chiliensis **SO**	**b**	**Peru coast**	**17/30**	**PS; (*17, 18, 67, 77*)‡; (*21*)§**
Sciaena deliciosa **SC**	**p**	**Chimbote, Peru**	**25/318**	**(*91– 93*), PS; (*87*)‡**
Sciaena deliciosa **SC**	**p**	**Trujillo coast**	**16/112**	**(*17, 18, 60, 62*)§**

Host species and family	Site	Location	Prevalence	Reference
***Sciaena deliciosa* SC**	**p**	**Ventanilla-Callao area**	**6/35**	**(*24*)† 26**
Sciaena callaensis SC	s, p	Trujillo coast	4/17	(*91*)
Scomber japonicus SO	v, p	Peru coast	ND	(*86*); (*21*)† 4; (*87*)† 48; (*89*)† 36
Scomberomorus maculatus SO	b	Peru coast	3/16	(*21*)§; (*87, 89*)†16
***Seriolella violacea* CE**	**p**	**Trujillo coast**	**3/56**	**(*87*); (*21*)† 4; (*91*)† 250**
***Trachinotus paitensis* CA**	**p**	**Trujillo coast**	**2/16**	**(*87*); (*21*)† 4; PS† 9**
***Trachurus murphyi*[4] CA**	**b**	**Chorrillos, Lima**	**8/20**	**PS; (*26, 33, 45, 47, 67, 77, 90*)‡; (*21*)† 37; (*87*)† 59; (*89*)† 34; (*91*)† 222**

*Bold text indicates fish hosts confirmed by DNA sequencing or experimental infections (*89*); AR, Ariidae; CA, Carangidae; CE, Centrolophidae; CO, Coryphaenidae; HA, Haemulidae; ME, Merlucciidae; MU, Mugilidae; ND, no data available; OP, Ophidiidae; PA, Paralichthyidae; SC, Sciaenidae; SO, Scombridae; TR, Triakidae; Site, site of infection; b, body cavity; I, intestinal surface; p, peritoneum; s, stomach surface; v, viscera; PS, present study.
†Plerocercoids were not found, followed with several dissected fish.
‡Anamnesis (case-patient–provided) data.
§Experimental infections of dogs and man failed (Baer et al. 1969).
¶Also reported as *Trachurus symmetricus murphyi* (see Froese & Pauly, 2015).

Technical Appendix Table 3. Historical survey of clinical cases of diphyllobothriosis caused by *Adenocephalus pacificusl*, 1961–2015*

Country	Age, sex	Symptoms	Treatment	Dietary habits	Reference
Peru	23, M	Grow fat†	Spontaneously	Fish	(*14*)
	47, F	A, D, V	Clorhidrat (acranyl)	ND	(*15*)
	4, M	None	Chloromycetin	P. peruanus	(*17*)
	14, F	A,F,N	After taking “weaver oil”	Cebiche	(*17*)
	21, M	None	Spontaneously	Cebiche	(*17*)
	7, F	None	Spontaneously	S. chilensis	(*17*)
	23, M	None	Spontaneously	Cebiche	(*17*)
	26, M	A,I, N,W	Spontaneously	ND	(*17*)
	30, F	H,N,W	Spontaneously	Marine fish	(*17*)
	23, M	A	Metroquin	Cebiche	(*18*)
	38, F	A,D	Metroquin	Several fish species	(*18*)
	24, F	A,W	Metroquin	Several fish species	(*18*)
	M	ND	Spontaneously	ND	(*19*)
	ND	25 cases, no details	Praziquantel	Cebiche	(*30*)
	ND	7 cases, no details	ND	Cebiche	(*31*)
	4–75	A, D, N, W	Praziquantel	ND	(*38*)
		None	ND	ND	(*38*)
	5, F	ND	ND	M. gayi	(*47*)
	2–18	A, D, V	Niclosamide	marine fish	(*67*)
	ND	None	Spontaneously	marine fish	(*67*)
	3–66	A, D, M, W	Niclosamide	Cebiche	(*77*)
		None	Spontaneously	Cebiche	(*77*)
	42, F	A, D, N, V	Praziquantel	Cebiche	(82)
Chile	6	A,N,V	Niclosamide	Cebiche	(*26*)
	29	A,N,M	Yomesan		(*27*)
	45, F	ND	ND	Raw sea fish	(*28*)
	23,M	ND	ND	Raw sea fish	(*28*)
	55, F	A,D,N	Niclosamide	*T. murphyi*	(*33*)
	35, M	A,D,N	Niclosamide	*T. murphyi*	(*33*)
	7, M	A,D,N	Niclosamide	*T. murphyi*	(*33*)
	43, F	A,D,N	Niclosamide	*T. murphyi*	(*33*)
	32, M	A,D,N	Niclosamide	*T. murphyi*	(*33*)
	53, M	A,D,N	Spontaneously	*T. murphyi*	(*33*)
	48, M	A,D,N	Spontaneously	*T. murphyi*	(*33*)
	45, F	A,N	Niclosamide	*T. murphyi*	(*33*)
	23, M	None	Niclosamide	*T. murphyi*	(*33*)
	40, M	None	Niclosamide	*T. murphyi*	(*33*)
	7, M	None	Niclosamide	*T. murphyi*	(*33*)
	3, M	None	Spontaneously	Smoked *T. murphyi*	(*45*)
	26, F	ND	Spontaneously	*Sciaena deliciosa*	(*60*)
	3, M	None	Spontaneously	Cebiche	(*62*)
	36, F	A	ND	Cebiche	(*74*)
Japan	54, M	ND	Spontaneously	Seaman	(*44*)
	59, M	None	Spontaneously	Sashimi	(*53*)
Spain	3	None	Spontaneously	ND	(*66*)
	50, M	None	Spontaneously	Raw fish	(*83*)
	52, F	None	Praziquantel	Raw fish	(*83*)
	27, M	None	Spontaneously	ND	(*84*)

* A, abdominal pain; D, diarrhea; F, female; H, male; ; I, intensive salivation in the morning;*M. gayi*, *Merluccius gayi peruanus*; M, megablastic anemia; N, nausea; ND, no data available; *P. peruanus*, *Paralonchurus peruanus*; *S. chilensis*, *Sarda chilensis*; Spontanously, spontaneous elimination of tapeworms from the patient; *T. murphyi*, *Trachurus murphyi;* V, vomiting; W, weight loss.
†The only change in the patient’s condition was described as “grow fat.” This term was not defined.

References

1. Stiles CW, Hasall A. Internal parasites of the fur seal, Chapter VII. In: Jordan DS, editor. The fur seals and fur-seal islands of the North Pacific Ocean. Washington D.C.: U. S. Government Printing Office; 1899. p. 629.

2. Nybelin O. Säugetier- und Vogelcestoden von Juan Fernandez. The natural history of Juan Fernandez and Easter Island. Uppsala; 1931. p. 493–523.

3. Drummond FH. 16. Cestoda: In Lady Julia Percy Island. Rep. Exped. McCoy Soc. field investigation and research. Proc R Soc Vic. 1937;49:401–4.

4. Johnston TH. Entozoa from the Australian hair seal. Proc Linn Soc N S W. 1937;62:9–16. http://www.biodiversitylibrary.org/page/34903721#page/59/mode/1up

5. Afanasev WPv. Parasitofauna of fish-eating mammals of Komandorskie Islands. Uchenie zapiski Leningradskogoi Gosudarstvennogo Universiteta [In Russian.]. Seria Biologii. 1941;74:93–117.

6. Wardle RA, McLeod JA, Stewart IE. Lühe's "*Diphyllobothrium*" (Cestoda). J Parasitol. 1947;33:319–30. PubMed http://dx.doi.org/10.2307/3273360

7. Stunkard HW. Pseudophyllidea cestodes from Alaskan pinnipeds. J Parasitol. 1948;34:211–28. PubMed http://dx.doi.org/10.2307/3273267

8. Yamaguti S. Studies on the helminth fauna of Japan. Part 47. Cestodes of marine mammals and birds. Acta Med Okayama. 1951;7:307–14. http://www.researchgate.net/publication/40008925_Studies_on_the_Helminth_Fauna_of_Japan.__Part_47._Cestodes_of_Marine_Mammals_and_Birds._With_2_Plates

9. Krotov AI, Delyamure SL. On the parasitic worms of mammals and birds of the USSR. Trudy GELAN. 1952;6:278–92.

10. Markowski S. The cestodes of pinnipeds in the Arctic and other regions. J Helminthol. 1952;26:171–214. http://dx.doi.org/10.1017/S0022149X00032612

11. Margolis L. List of the parasites recorded from sea mammals caught off the West coast of North America. J Fish Res Bd Can. 1954;11:267–83. http://dx.doi.org/10.1139/f54-017

12. Delyamure SL. Helminthofauna of marine mammals (ecology and phylogeny). Moscow: Akademia Nauk SSSR; 1955. http://www.worldcat.org/title/helminthofauna-of-marine-mammals-ecology-and-phylogeny/oclc/630124726

13. Margolis L. Parasitic helminths and arthropods from Pinnipedia of the Canadian Pacific coast. J Fish Res Bd Can. 1956;13:489–505. http://dx.doi.org/10.1139/f56-030

14. Miranda H, Fernandez W, Castillo A, Soriano M. Presencia en Trujillo Perú del *Diphyllobothrium latum* (Linneo, 1758) Luhe, 1910. Arch Peru Pat Clin. 1961;15:67–76.

15. Castillo Pretell F, Arrasco Seclén M, Ibánez Herrera N. Sobre un caso de diphyllobothriasos procedente de la ciudad de Chiclayo – Peru. Trabajo presentado a la Jornada de Microbiologia y Parasitologia de Trujillo; 1962. p. 51.

16. Keyes MC. Pathology of the northern fur seal. J Am Vet Med Assoc. 1965;147:1090–5. PubMed

17. Baer JG, Miranda CH, Fernandez RW, Medina TJ. Human diphyllobothriasis in Peru. Z Parasitenkd. 1967;28:277–89. PubMed http://dx.doi.org/10.1007/BF00260267

18. Miranda H, Fernandez W, Bocanegra R. Diphyllobothriasis, estado actual en el Perú. Descripción de nuevos casos. Arch Peru Patol Clin. 1967;21:53–70.

19. Rêgo AA. Uma nova ocorrência de „*Lüheella*“ (Diphyllobothriidae, Pseudophyllidea) em homem. Atas Soc Biol Rio de Janeiro. 1967;10:161–2.

20. Delyamure SL, Parukhin AM. A new *Diphyllobothrium* – parasite of the South-African fur-seal. [In Russian.]. Biol Morya. 1968;14:25–33.

21. Baer JG. *Diphyllobothrium pacificum*, a tapeworm from sea lions endemic in man along the coastal area of Peru. J Fish Res Bd Can. 1969;26:717–23. http://dx.doi.org/10.1139/f69-071

22. Machida M. Parasites of the northern fur seal and their relationship to the Breeding Islands. Proc Jpn Soc Syst Zool. 1969;5:16–7.

23. Dailey MD, Hill JE. A survey of metazoan parasites infecting the California (*Zalophus califorianus*) and Steller (*Eumetopias jubatus*) sea lion. Bull South Calif Acad Sci. 1970;69:126–32. http://biodiversitylibrary.org/page/34157615#page/988/mode/1up

24. Chupakhina TI. The helminth fauna of the fur seal on Robben Island. [In Russian.]. Trudy AtlantNIRO. 1971;39:166–70.

25. Kovalenko LM. Helminth fauna of Otariidae inhabiting the Kuril Islands. In: Marine mammals: proceedings of the VI All-Union Council on study of marine mammals (Kiev, October, 1975). vol. 1. Kiev: Naukova Dumka; 1975. p. 137–139. [In Russian.]

26. Atias A, Cattan P. Primer caso humano de infección por *Diphyllobothrium pacificum* en Chile. Rev Med Chil. 1976;104:216–7. PubMed

27. Cristoffanini AP, Ibarra H, Vega I, Martínez A, Bertoglio JC. *Diphyllobothrium latum* induced megaloblastic anemia. Rev Med Chil. 1976;104:921–4. PubMed

28. Sagua H, Miranda E, Fuentes A, Vladillo V. *Diphyllobothrium pacificum* (Nybelin, 1931), Margolis 1956. Primeros dos casos de infección humana en el norte de Chile. Bol Chil Parasitol. 1976;31:33. PubMed

29. Cattan PE, Atias A, Babero BB, Torres D. Helmintofauna de Chile. V. Primer hallazgo de *Diphyllobothrium pacificum* (Nybelin 1931) Margolis 1956, en lobos marinos de la costa chilena. Rev Iber Parasitol. 1977;37:285–90.

30. Espejo H. Treatment of infections by *Hymenolepis nana, Taenia saginata, Taenia solium* and *Diphyllobothrium pacificum* with praziquantel (Embay 8440). Bol Chil Parasitol. 1977;32:39–40. PubMed

31. Guerrero VHM. Infestación por cestodes de los generos *Taenia* y *Diphyllobothrium* en humanos de la provincia de Chiclayo. Universidad (UNPRG Lambayeque-Perú). 1977;1:3–5.

32. Stroud RK. Parasites and associated pathology observed in pinnipeds stranded along the Oregon coast. J Wildl Dis. 1978;14:292–8. PubMed http://dx.doi.org/10.7589/0090-3558-14.3.292

33. Sagua H, Fuentes A, Soto J, Delano B. Human diphyllobotriasis due to *Diphyllobothrium pacificum* in Chile. An experience with 11 cases. Rev Med Chil. 1979;107:16–9. PubMed

34. Cattan PE, Yanez JL, Torres D. Helmintos parásitos del lobo fino *Arctocephalus philippii* (Peters, 1866) de Juan Fernández. Bol Chil Parasitol. 1980;35:73–5. PubMed

35. George-Nascimento M, Carvajal J. Helminth parasites of the South American sea lion *Otaria flavescens* from the Gulf of Arauco, Chile. Bol Chil Parasitol. 1981;36:72–3. PubMed

36. Maejima J, Yazaki S, Fukumoto S, Hiraga M, Kamo H. Morphological observations of *Diphyllobothrium pacificum* (Nybelin, 1931) Margolis, 1956 from fur seals, *Callorhinus ursinus* in Japan. Yonago Acta Med. 1981;25:69–79.

37. Kamo H, Maejima J, Yazaki S, Otsuru M, Hesegava H, Kuniyoshi S, et al. Occurrence of human infection with *Diphyllobothrium pacificum* (Nybelin, 1931) Margolis, 1956 in Japan. Kisechugaku Zasshi. 1982;31:165–70.

38. Lumbreras H, Terashima A, Alvarez H, Tello R, Guerra H. Single dose treatment with praziquantel (Cesol R, EmBay 8440) of human cestodiasis caused by *Diphyllobothrium pacificum.* Tropenmed Parasitol. 1982;33:5–7. PubMed

39. Patrucco R, Tello R, Bonavia D. Parasitological studies of coprolites of pre-hispanic Peruvian populations. Curr Anthropol. 1983;24:393–4. http://dx.doi.org/10.1086/203016

40. Torres P, Figueroa L, Franjola R. Pseudophyllidea in the South of Chile. IX. Types of plerocercoids in trouts from five lakes and new cases of *Diphyllobothrium latum* in man and *D. pacificum* in a dog. Int J Zoonoses. 1983;10:15–21. PubMed

41. Ferreira LF, de Araújo AJG, Confalonieri UEC, Nunez L. The finding of eggs of *Diphyllobothrium* in human coprolites (4,100–1,950 B.C.) from northern Chile. Mem Inst Oswaldo Cruz. 1984;79:175–80. PubMed http://dx.doi.org/10.1590/S0074-02761984000200004

42. Shults LM. Helminth parasites of the Steller sea lion, *Eumetopias jubatus*, in Alaska. Proc Helminthol Soc Wash. 1986 1986;53:194–7.

43. Yurakhno MV, Taikov IM. Some preliminary results of parasitological autopsies of commander seals in 1984–1985. In: Study, protection and management of marine mammals. Proceedings of the 9th All-Union Conference, Arkhangelsk; 1986. p. 435–436. [In Russian.]

44. Makiya K, Tsukamoto M, Horio M, Goto M. *Diphyllobothrium pacificum*, a cestode of marine mammals, expelled from a Japanese seaman. Kisechugaku Zasshi. 1987;36:145–53.

45. Mercado R, Torres P. A. L, Schenone H. Infección por *Diphyllobothrium pacificum,* probablemente adquirida en el sur de Chile por un niño de tres años. Bol Chil Parasitol. 1988;43:54–6. PubMed

46. Bester MN. Endoparasites of the subantarctic fur seal *Arctocephalus tropicalis* from Gough Island. S Afr J Zool. 1989;24:363–5.

47. Gárate I, Naupay A. Un caso de diphyllobotriosis en zona altoandina. Rev Peru Parasitol. 1990;14:84–6.

48. Yazaki S, Fukumoto S, Maejima J, Miyahara M. Comparative observations of *Diphyllobothrium pacificum* from a man and from fur seals. J Yonago Med Assoc. 1990;41:204–10.

49. Pansegrow HM. A taxonomic and quantitative study of the endoparasites of the South African fur seal, *Arctocephalus pusillus pusillus*, Schreber, with comments on their life-histories and veterinary and medical importance. Master Thesis, University of Stellenbosch, Stellenbosch. 1990.

50. Gallegos R, Brousselle C. Intestinal parasitosis of inhabitants living in an Ecuatorian archipelago [in French]. Bull Soc Fr Parasitol. 1991;9:219–23. http://cat.inist.fr/?aModele=afficheN&cpsidt=5077192

51. Gage LJ, Gerber JA, Smith DM, Morgan LE. Rehabilitation and treatment success rate of California sea lions (*Zalophus californianus*) and northern fur seals (*Callorhinus ursinus*) stranded along the central and northern California coast, 1984–1990. J Zoo Wildl Med. 1993;24:41–7. http://www.jstor.org/stable/20460312

52. Tantaleán VM. Algunos helminthos de mamíferos marinos del Perú y su importancia médica [in Spanish]. Rev Peru Med Trop. 1993;7:67–71. http://bases.bireme.br/cgi-

bin/wxislind.exe/iah/online/?IsisScript=iah/iah.xis&base=LILACS&lang=p&nextAction=lnk&exprSearch=154651&indexSearch=ID

53. Tsuboi T, Torii M, Hirai K. Light and scanning electron microscopy of *Diphyllobothrium pacificum* expelled from a man. Jap J Parasitol. 1993;42:422–8.

54. Huiza A, Quiñones A, Rojas CY, Leandro M. Enteroparasitosis en el sector 23 A. Primera etapa del distrito de Villa El Salvador – Lima. Marzo–Abril, 1993. Rev Per Med Trop. 1994;8:75–80. http://sisbib.unmsm.edu.pe/BVRevistas/rpm_trop/v08_n1-2/Pdf/a13.pdf

55. Luna A, Zamora A, Santa María L. Prevalencia, distribución e intensidad de infección de parasitosis intestinales en escolares del primer grado de primaria de San Juan de Miraflores – Lima – 1993 [in Spanish]. Rev Per Med Trop. 1994;8:75–80.

56. Liñan-Abanto R, Jara CA. Frecuencia y aspectos epidemiológicos del parasitismo por helmintos intestinales en la población infantil de Paiján, La Libertad – Perú. Rev Peru Parasitol. 1995;11:46–50.

57. Montenegro TC, Miranda EA, Gilman R. Production of monoclonal antibodies for the identification of the eggs of *Taenia solium.* Ann Trop Med Parasitol. 1996;90:145–55. PubMed

58. Stewardson CL, Fourie HJ. Endoparasites of the Cape fur seal *Arctocephalus pussilus pussilus* from the Eastern Cape coast of South Africa. Trans R Soc S Afr. 1998;53:33–51. http://dx.doi.org/10.1080/00359199809520372

59. Yurakhno MV. Diseases and Parasites. In: Sokolov, V.E., Aristov, A.A., Lisitzina, T.U. (Eds.), The northern fur seal. Systematics, morphology, ecology, behavior. Nauka, Moscow, Russia; 1998. p. 810–899. [In Russian.]

60. González A, Sagua H, Cortés L, Lobo J, Neira I, Araya J. *Diphyllobothrium pacificum* human infection. Report of one case. Rev Med Chil. 1999;127:75–7. PubMed

61. Terashima AI. Diphyllobothriasis. Diagnostico. 2000;39:1–4.

62. Sagua HF, Aliaga PR, Neira CI, Araya RJ, González CJ. Diphyllobothriosis humana por infección por *Diphyllobothrium pacificum* en un niño de 3 años en Antofagasta, Chile. Rev Chil Pediatr. 2000;71:427–9. http://dx.doi.org/10.4067/S0370-41062000000500009

63. Cabrera RC, Tantaleán MV, Rojas RM. *Diphyllobothrium pacificum* (Nybelin, 1931) Margolis, 1956 en *Canis familiaris* de la ciudad de Chincha, Perú. Bol Chil Parasitol. 2001;56:1–2. PubMed http://dx.doi.org/10.4067/S0365-94022001000100007

64. Sagua H, Neira I, Araya J, González J. Nuevos casos de infección humana por *Diphyllobothrium pacificum* (Nybelin, 1931) Margolis, 1956 en Chile y su probable relación con el fenómeno de El Niño, 1975–2000. Bol Chil Parasitol. 2001;56:22. PubMed

65. Anonymous. Helmintos intestinales en el Perú: análisis de la prevalencia (1981–2001). Lima: Ministerio de Salud, Peru; 2003.

66. Colomina J, Villar J, Esteban G. Asymptomatic infection by *Diphyllobothrium latum* in a Spanish 3-year-old child. Med Clin (Barc). 2002;118:279. PubMed http://dx.doi.org/10.1016/S0025-7753(02)72359-2

67. Medina Flores DJ, Tantaleán VM, León RM, Cano RM. *Diphyllobothrium pacificum* en niños del Perú. Diagnostico. 2002;41.

68. Holiday DM, Guillen S, Richardson DJ. Diphyllobothriasis of the Chiribaya culture (700–1476 AD) of southern Peru. Comp Parasitol. 2003;70:167–71. http://dx.doi.org/10.1654/4096

69. Martinson E, Reinhard KJ, Buikstra JE, de la Cruz KD. Pathoecology of Chiribaya parasitism. Mem Inst Oswaldo Cruz. 2003;98:195–205. PubMed http://dx.doi.org/10.1590/S0074-02762003000900029

70. Reinhard K, Urban O. Diagnosing ancient diphyllobothriasis from Chinchorro mummies. Mem Inst Oswaldo Cruz. 2003;98:191–3. PubMed http://dx.doi.org/10.1590/S0074-02762003000900028

71. Santoro C, Vinton SD, Reinhard KJ. Inca expansion and parasitism in the Lluta Valley: preliminary data. Mem Inst Oswaldo Cruz. 2003;98(Suppl. 1):161–3. PubMed http://dx.doi.org/10.1590/S0074-02762003000900024

72. García C, Rodríguez E, Do N, López de Castilla D, Terashima A, Gotuzzo E. Parasitosis intestinal en el paciente con infección VIH-SIDA. Rev Gastroenterol Peru. 2006;26:21–4. PubMed

73. Morgades D, Katz H, Castro O, Capellino D, Casas L, Benítez G, et al. Fauna parasitaria del lobo fino *Arctocephalus australis* y del león marino *Otaria flavescens* (Mammalia, Otariidae) en la costa uruguaya. In: Menafra R, Rodríguez-Gallego L, Scarabino F, Conde D, editors. Bases para la Conservación y el Manejo de la Costa Uruguaya. Montevideo: Vida Silvestre Uruguay; 2006. p. 89–96.

74. Mercado R, Yamasaki H, Kato M, Munoz V, Sagua H, Torres P, et al. Molecular identification of the *Diphyllobothrium* species causing diphyllobothriasis in Chilean patients. Parasitol Res. 2010;106:995–1000. PubMed http://dx.doi.org/10.1007/s00436-010-1765-6

75. Rausch RL, Adams AM, Margolis L. Identity of *Diphyllobothrium* spp. (Cestoda: Diphyllobothriidae) from sea lions and people along the Pacific coast of South America. J Parasitol. 2010;96:359–65. PubMed http://dx.doi.org/10.1645/GE-2257.1

76. Casquina-Guere L, Martínez-Barrios E. Prevalencia y epidemiología del parasitismo intestinal en escolares de nivel primario de Pucchún, Camaná, Arequipa, Perú, 2006. Neotrop Helminthol. 2011;5:247–55.

77. Jiménez JA, Rodriguez S, Gamboa R, Rodriguez L, Garcia HH. *Diphyllobothrium pacificum* infection is seldom associated with megaloblastic anemia. Am J Trop Med Hyg. 2012;87:897–901. PubMed http://dx.doi.org/10.4269/ajtmh.2012.12-0067

78. Richardson DJ, Guillén S, Beckett R, Kyle W, Conlogue G, Harper-Beckett K. Archaeohelminthology of the Chiribaya Shepherd, *Canis familiaris* (700–1476 a.d.) from southern Peru. Comp Parasitol. 2012;79:133–7. http://dx.doi.org/10.1654/4490.1

79. Hernández-Orts JS, Montero FE, Juan-García A, García NA, Crespo EA, Raga JA, et al. Intestinal helminth fauna of the South American sea lion *Otaria flavescens* and fur seal *Arctocephalus australis* from northern Patagonia, Argentina. J Helminthol. 2013;87:336–47. PubMed http://dx.doi.org/10.1017/S0022149X12000454

80. Le Bailly M, Bouchet F. *Diphyllobothrium* in the past: review and new records. Int J Paleopathol. 2013;3:182–7. http://dx.doi.org/10.1016/j.ijpp.2013.05.004

81. Reinhard KJ, Ferreira LF, Bouchet F, Sianto L, Dutra JMF, Iniguez A, et al. Food, parasites, and epidemiological transitions: a broad perspective. Int J Paleopathol. 2013;3:150–7. http://dx.doi.org/10.1016/j.ijpp.2013.05.003

82. Villena O, Kianman W, Núñez J, Alcántara A, Villena A. Pancreatitis aguda por *Diphyllbothrium pacificum*. Enfermedades del Aparato Digestivo. 2001;5:40–1.

83. Pastor-Valle J, González LM, Martín-Clemente JP, Merino FJ, Gottstein B, Gárate T. Molecular diagnosis of diphyllobothriasis in Spain, most presumably acquired via imported fish, or sojourn abroad. New Microbes New Infect. 2014;2:1–6. PubMed http://dx.doi.org/10.1002/2052-2975.28

84. Kuchta R, Esteban J-G, Brabec J, Scholz T. Misidentification of *Diphyllobothrium* species related to global fish trade, Europe. Emerg Infect Dis. 2014;20:1955–7. PubMed http://dx.doi.org/10.3201/eid2011.140996

85. Hernández-Orts JS, Kuchta R, Kuzmina T, Brabec J, Scholz T. Unexpected morphological plasticity and global geographical distribution of the Pacific human tapeworm *Adenocephalus pacificus* (syn. *Diphyllobothrium pacificum*): molecular and morphological survey. Acta Trop. 2015; 149: 168–78.. PubMed http://dx.doi.org/10.1016/j.actatropica.2015.05.017

86. Luque J. Formas larvarias de helmintos en peces marinos del Peru. Parasit al Día. 1991;15:43–8.

87. Escalante H, Miranda H. *Diphyllobothrium pacificum*: hallazgo de larvas plerocercoides en peces marinos del Perú y desarrollo de formas adultas del parásito en *Canis familiaris.* Bol Chil Parasitol. 1986;41:7–13. PubMed

88. Tantalean VM, Carvajal CG, Martinez RR, Huiza FA. Helmintos parasites de peces marinos de la Costa peruana. NCTL Ser Div Cient. 1982; 1.

89. Escalante H. Plerocercoid larvae of Diphyllobothriidae Lühe, 1910: finding in Peruvian sea fish for human consumption. Bol Chil Parasitol. 1983;38:50–2. PubMed

90. Tantaleán MV, Huiza AF. Sinopsis de los parásitos de peces marinos de la Costa Peruana. Biotempo. 1994;1:53–101.

91. Tantaleán MV. The finding of plerocercoid larvae of Diphyllobothriidae Lühe, 1910 (Cestoda) in Peruvian sea fish. Bol Chil Parasitol. 1975;30:18–20. PubMed

92. Llerena CZ, Chávez VA, Casas AE. Frequency of Diphyllobothriidae and Anisakidae larva in commercial marine fish sold in the port of Callao. Rev Investig Vet Peru. 2001;12.

93. Chero J, Sáez G, Iannacone J, Aquino W. Ecological aspects of parasitic helminths of lorna drum *Sciaena deliciosa* (Tschudi, 1846) (Perciformes: Sciaenidae) acquired at the fishing terminal of Ventanilla, Callao, Peru. Sinopsis de los parásitos de peces marinos de la Costa Peruana. Neotrop Helminthol. 2014;8:59–76. http://sisbib.unmsm.edu.pe/bvrevistas/neohel/v8n2/contenido.htm

94. Ayulo V, Filomena C. Incidencia del parasitismo en los niños de la costa, sierra y selva del Peru. V Congreso Panamericano de Pediatría. Ago 5–11 1957; Lima; 1957. p. 463–79.
